# Risk perceptions, government role, and public protective behavior in the face of environmental health hazards

**DOI:** 10.1016/j.nexres.2026.101537

**Published:** 2026-03-03

**Authors:** Rotem Dvir, Hyunseok Hwang

**Affiliations:** a Institute for Science, Technology and Public Policy, The Bush school of Government and Public Service, Texas A&M University, USA; b Department of Sociology, Ajou University South Korea

**Keywords:** Environmental health hazard, Protection motivation theory, Mitigation behavior, Risk perceptions, Government competence, Pollution

## Abstract

In this study, we expand research on climate change mitigation behavior by exploring individuals’ protective actions in the face of a diverse set of environmental hazards including air pollution, water and soil contamination, spillage of toxicants, polluted food and consumer products. Also, we investigate protective actions related to consumption choices (food and cleaning products) or behavioral change (staying indoors). Those actions reflect a broader range of responses of at-risk individuals who wish to reduce the health-related ramifications of pollution-type hazards. We build on *Protection Motivation Theory (PMT)* to describe the factors that motivate individuals’ behavior. In particular, we emphasize the emergence of risk perceptions and their subsequent effects on behavior. We also account for PMT’s coping appraisal by assessing the role of the government in motivating protective actions. We use American national-level data from a survey that highlights environmental health and employ both regression and structural equation modeling (SEM). The empirical analysis reveals the critical role of risk perceptions that have both a direct effect on mitigation behavior and it mediates the effects of several antecedents on protective actions. Our findings extend research on individual protective behavior to mitigate the detrimental health effects of less common hazards like air and water pollution or soil contamination and toxicants release. In particular, we offer evidence on the drivers of mitigating actions to reduce the health-specific risks from environmental hazards that are not necessarily natural and can be a result of human action.

## Introduction

On February 3rd, 2023 a train carrying hazardous chemicals derailed in eastern Ohio causing severe local health risks. Authorities evacuated several nearby at-risk communities, and encouraged locals to engage in protective actions such as frequent in-door cleaning and monitoring air quality.^1^ Similarly, the 2023 summer wildfires that engulfed Canada and the northern US led health experts to issue advisory calls for staying indoors, ensuring air screening and monitoring health conditions.^2^ Environmental hazards (both man-made and natural) create a host of health risks to local communities, and other than solutions implemented by authorities, individuals can engage in multiple protective actions to limit their exposure and reduce health threats.

In this study, we explore individuals’ protective behavior facing environmental hazards and focus on two under-studied aspects. First, we emphasize hazards such as air pollution, water and soil contamination, spillage of toxic materials, polluted consumer goods, etc. These may result from intentional or unintentional human actions. Also, while those are not natural hazards, they can result from extreme weather events like floods or excessive heat, and their consequences are just as bad. Second, we investigate health-oriented actions undertaken by individuals at-risk. Rather than focus on preparedness or the broader pro-environmental behavior, we explore responses that are intended to reduce the health-related ramifications individuals face from pollution or contamination-type environmental hazards.

We utilize *Protection Motivation Theory (PMT)* [1,2] to describe the factors that drive individuals’ actions. In particular, we explore risk appraisal (a central tenant of PMT) and emphasize how the emergence of risk perceptions shape subsequent protective behavior. Then, we assess a more unique (and understudied) angle of PMT’s second element, the coping appraisal, by accounting for views about authorities’ competence and capacity to reduce the risks, and how it affects individuals’ decisions to adopt protective actions.

Using national-level data collected with a survey that emphasizes issues of environmental health, we test multiple factors that influence Americans’ protective behavior. The results reveal the powerful role of PMT’s risk appraisal (perceptions) in motivating mitigation behavior as well as mediating the effects of individual-level antecedents (i.e., awareness, knowledge, and health conditions). At the same time, we find that coping appraisal, conceptualized as perceived government competence, is not associated with mitigation. Overall, our findings extend knowledge on individual protective behavior, and explore broader aspects of PMT in the context of environmental hazards. In particular, we offer evidence on the drivers of a diverse set of mitigating actions to reduce the health-specific risks from hazards that are not necessarily natural, and in many cases are a function of human intervention.

### Mitigation of environmental hazards

Research on environmental hazards discusses two broad approaches to mitigating their adverse consequences. At the societal level, governments enact different policies designed to reduce the negative impacts of disasters [3–7]. However, individuals also engage in multiple mitigating or adaptive actions^3^ to reduce damages to their health. Studies have built on health behavior theories like the health belief model (HBM) that explain the factors that motivate individuals to engage in actions like vaccination [8,9]. Researchers used a similar logic to describe *Pro--environmental behavior (PEB)*: different actions that individuals adopt in response to climate change or environmental threats, and are intended to reduce harm to themselves or the environment [10–13].

Within the context of environmental hazards, studies explore natural disasters and assess individuals’ preparedness or post-event reactions [14]. A central objective of this research area is to identify the correlates of mitigating behavior, and find ways to encourage people to engage in actions that would lessen adverse health effects [15].

A smaller volume of studies looks into behavior in the face of hazards that are not necessarily natural and result from pollution or contamination events. Zhao et al. [16] explore individuals’ willingness to purchase energy saving products to counter air pollution threats. Similarly, authors study the drivers of protective actions like wearing a mask or limiting outdoor activities during haze pollution or smog [17–19]. Recent work investigates individuals’ awareness and threat perceptions of marine pollutants [20,21] or microplastics [22]. The behavioral angle is studied within the context of pro-environmental behavior including less plastics usage [23–25], and altering outdoor activities, the consumption of seafood or drinking water due to water pollution threats [26].

The research on pollution-type hazards is less common, with most studies emphasizing different explanations to observed behavior [18, 26], and some work rely on clear framework like the *Theory of planned Behavior* [16]. We extend this approach by offering a theoretically driven perspective on the factors that motivate protective behavior to reduce the detrimental health risks associated with exposure to various contamination and pollution hazards. In particular, we draw on *Protection Motivation Theory (PMT)*, a theoretical framework widely used to understand protective behaviors, to examine which factors predict behavior aimed at safe-guarding the health of individuals facing a diverse set of environmental hazards.

### Environmental health hazards and individual protective behavior

#### Protection Motivation Theory (PMT)

[1,2] is a prevalent framework that explains why individuals engage in actions versus different threats. The theory stipulates that individuals partake in protective behavior based on their appraisal (or perception) of risk and coping ability. *Risk appraisal*, referred as risk perception in this study, encompasses individuals’ expectation of the hazard’s probability, severity, and the relevant degree of concern. *Coping appraisal* is an assessment of our capacity to engage in risk prevention behavior or the efficacy of the coping response [1,27].

PMT is a widely applied theoretical framework to describe the factors that drive protective behavior in the face of environmental hazards [27–29]. In their review of the literature using PMT, Kothe et al. [30] identify that most studies address climate change broadly, use data from certain non-random populations (students, farmers, etc.), and test a limited set of specific mitigation actions. More in-line with our work, Little et al. [31] use PMT to investigate the factors that drive protective behavior facing toxic water pollution in three American states. Recent work implements PMT to study protective practices among farmers in Nepal facing risks from pesticides exposure [32].

Research on hazards like pollution and contamination remain relatively limited, and only a few studies have applied PMT in this context. Our work adds to this limited stream of studies with a conceptual model that describes a more unique angle of PMT by studying one of the theory’s main pillars (coping appraisal) in a different way. Our conceptualization diverges from most PMT studies as we adjust some of the theory’s elements to fit the context of pollution-type hazards. In particular, we posit that due to the nature of pollution-type hazards (potential exposure to dangerous materials), mitigation requires a specialized and expert intervention. Thus, it is possible that people’s trust in the capacity of governments to address the threat is more important than their self-efficacy. In addition, our study extends the application of PMT in the context of environmental hazards in two ways: (1) existing work investigating pollution hazards tend to focus on one or two specific threats. Our study moves beyond the conventional focus on air and water pollution to examine a broader range of hazards, including soil contamination, toxicants release, treated wastewater, hazards in consumer products and others. Testing an extensive list of threats offers insights about the role of the different factors in mitigation efforts of multiple environmental threats; (2) We analyze diverse types of protective actions including consumption and cleaning behavior or limiting outdoor activities. Analyzing the predictors of a larger set of actions offers implications for how we can motivate individuals to protect themselves against a wider range of health-related threats from environmental hazards.

#### Risk perceptions (Appraisal)

The concept of risk perceptions describes the process in which we interpret different signals from various sources and how we form subjective judgments about threats [33,34]. Therefore, the more people view hazards as representing a risk for their well-being, the more likely they are to engage in protective behavior [35].

Research on environmental hazards shows that risk perceptions play either a direct or indirect role in motivating protective actions. Xie et al. [36] extend the climate change risk perception model (CCRPM) [37] and find that risk perceptions have a mediating effect on the willingness of respondents to engage in mitigation. Others demonstrate that risk perceptions are essential to the adoption of protective measures [18, 38–40]. Recently, Zhou et al. [41] demonstrate that high risk perceptions due to soil pollution motivated farmers to reduce the usage of plastics and dangerous pesticides. Using the lenses of PMT, a study in Thailand finds that threat appraisal of pollutants from waste disposal explains increased recycling [42]. Kothe et al. [30] broad review of studies that use PMT and environmental hazards find that, for the most part, threat appraisal is associated with increases in the intention to engage in protection behavior like water conservation, green consumerism and more.

Despite the relatively limited research on hazards such as water and soil contamination or toxicants, studies do address risk perceptions and show it motivates mitigation activities such as recycling and reduced plastics usage [43,44] or supporting broader government regulations of plastic waste and contamination sources [23,24]. Little et al. [31] explore local water pollutant threats among US residents and find that risk appraisal has a critical role in adopting protective behaviors. Others have shown that threat appraisal is essential in health protection actions in regions with high exposure to lead contamination [45].

Many studies that explore individual mitigation behavior contend that risk perceptions represent one stage in a sequential process [14,34, 36,46]. In our model, the focus is protective actions that reduce the health risks from environmental hazards, and thus risk perceptions directly shape behavior. At the same time, our model also describes several factors that underpin risk perceptions and serve as antecedents within the overall framework [19].

#### Awareness

The concept of awareness reflects individuals’ beliefs or general concerns about issues of environmental health and climate change. Greater concern indicates heightened recognition of the threats posed by hazards such as air pollution or toxic spills, which in turn increases the likelihood of adopting protective actions. Research on natural hazards has shown that a strong belief in threats from climate change increase risk perceptions [47–49] and that motivates adaptive behavior [46]. Akerlof et al. [50] find that awareness in the sense of belief in climate change and its implications is a strong predictor of health-related risk perceptions of environmental hazards. Some environmental scholars view awareness as a holistic concept that describes an evaluative feeling. The evidence suggests that this awareness-affect is one of the strongest predictors of climate change risk perceptions [37,51].

With respect to pollution-type hazards, work on microplastics in Germany has shown that awareness is a critical factor in the formation of risk perceptions [22,52]. A recent study of American public attitudes finds that awareness of pollution threats affects individuals’ risk assessment of such hazards [53]. Wu et al. [44] explore social media data in China and show how awareness about microplastics affects citizens’ health risk perceptions, and willingness to reduce plastic usage. In studies of marine and coastal pollution, scholars view awareness as an essential factor that influences risk attitudes, and the willingness to adopt mitigation behavior [54]. Our main proposition in this context is that awareness of general health threats from environmental hazards is associated with increases in risk perceptions.

#### Knowledge

Heightened risk perceptions that increase the willingness to initiate actions to reduce threats are also a function of knowledge. Research on pro-environmental behavior has shown that having relevant knowledge and understanding the benefits of mitigation is associated with actions [55,56]. Yet, Van Valkengoed and Steg [14] found these direct effects to be rather small. A larger volume of work shows that knowledge is essential to the emergence of environmental risk perceptions [36,51, 57], and in turn motivate pro-environmental behavior [46].

In studies of risks from hazards such as plastics or soil contamination, evidence suggest that knowledge, i.e. having a better grasp of hazards and their health implications, is important predictor of risk evaluations [58]. In turn, such attitudes motivate protective action [31,41,52]. At the same time, some scholars suggest that higher degree of knowledge reflects improved sense of control and therefore lower risk perceptions. Deng et al. [23] find such effects in China with microplastics. We expect knowledge to have a consistent effect on risk perceptions as it reflects individuals’ understanding of the hazards and potential methods to reduce the risks.

#### Experience

The literature of climate change and environmental hazards places an emphasis on experience [59]. The experience of events makes it more salient, and thus affects attitudes and the willingness to act [34,57,60, 61]. At the same time, Bradley et al. [46] identified a negative association between exposure and risk attitudes and suggested it may indicate that experience increases citizens’ resilience.

Our view of experience emphasizes health issues and their relationship with environmental hazards. In particular, we assess existing or past health conditions and their role in driving risk perceptions and behavior. We expect individuals with existing health problems to display higher levels of risk perceptions. Wei et al. [19] adopt a similar approach studying smog experience and its role in shaping risk perceptions and protective actions (see also [62]).

#### Coping appraisal

This study builds on the logic of PMT to explain protective behavior. Therefore, the decision to engage in mitigating actions is also a function of individuals’ assessment of their capacity to act, and how it would reduce risks. Studies suggest that this assessment includes aspects of self-efficacy and response efficacy [63]. In the context of natural hazards, evidence indicates that response efficacy is critical in motivating protective actions [46,64,65].

Studies using PMT to explore pollution-type threats show that coping appraisal, measured as self-efficacy or response efficacy, matter for protective behavior [30]. Little et al. [31] work on water pollutants find that response efficacy is critical for adopting health protective measures.^4^

We expand the role of this element by offering a different perspective on coping appraisal with respect to pollution or contamination hazards. Instead of conceptualizing coping appraisal as self- or response efficacy, we propose that considering the potential wide-scale damages from such hazards, public views of the role or the efficacy of actions taken by the government or local authorities are more relevant than each person’s individual capacity to deal with the threat. Considering the potential exposure to chemicals and other dangerous substances, expert handling and management of the response are critical. Thus, when facing air pollution, soil and water contamination or toxicants spillage, members of affected communities are more likely to take protective actions if they believe that the centralized response (local government) is insufficient or less effective [59]. In other words, it is the efficacy of actions implemented by local governments (and its competence in addressing the threats) that drive the decision to act, not views about their own efficacy in mitigating the threat.

The logic of our approach builds on work on trust in the context of healthcare. In particular, studies of the relationship between physicians and patients suggest that trust is essential to addressing risks when individuals face knowledge deficits. Patients need to trust the professionals who help them manage and address the risks to their health [66]. These insights can be extrapolated to the role of institutional trust - trust in authorities which manage health risks from environmental hazards like pollution, and thus affect subsequent behavioral choices. Within PMT, coping is mostly conceptualized as self-efficacy. Our approach allows to test another variant of efficacy (government trust/competence) and how it may influence behavior facing hazards that the government is qualified to address. This approach also proposes a possible integration of insights from health sciences with the logic of PMT in the context of environmental health hazards like pollution.

Most research on views of the government’s role discuss the concepts of competence or institutional trust and how they impact support for policies [4,67,68]. Studies that test how trust in government relates to individual actions offer mixed results. When exploring climate change actions, trust has been essential in motivating citizen participation [69, 70]. On the other hand, Arjomandi et al. [64] study water conservation and show that trust in governing institutions has a negative effect on engaging in protective action.

A limited number of studies of pollution and contaminations account for different versions of this factor. Kim et al. [25] explore individual and government mitigation of air pollution and find that trust in government is not associated with individual actions. Wei et al. [19] uses the concept of responsibility (defined as stakeholders who are responsible to protect citizens from hazards) and finds that this factor is not associated with intentions to adopt protective measures.

Considering these findings and within the confines of our protective behavior model, we propose that: (1) positive views on the efficacy of government actions or its competence are likely to reduce the tendency to take protective action; (2) the coping appraisal is likely to have a very limited effect on behavior.

The last set of factors we account for in our model is individual demographic characteristics. Beginning with correlates of risk perceptions, age is less consistent as younger people tend to be more concerned about climate change, but some evidence show that older respondents display increased risk perception of pollution-type hazards [20,22]. A more consistent finding is that women tend to display higher risk perceptions [20,22]. Education is another central factor and is usually negatively associated with risk perceptions. Since higher income and education are linked to greater sense of control, we expect it to reduce risk perceptions [71].

The effect of socio-demographic factors on behavior is less clear. Research suggest that age and gender are important drivers of pro-environmental behavior [72]. Also, individuals with higher income have less restrictions on actions like purchasing organic food or optimizing waste disposal [16].

### Data & methods

We test our model with data of a national representative sample collected between October 29th - November 18th, 2021. The sample is drawn from Ipsos’ web-enabled KnowlegePanel^®^, a probability-based panel designed to be representative of the U.S. population. The sampling strategy uses random selection of telephone numbers and residential address (ABS method) to invite respondents to the opt-in online panel. Participants also complete demographic survey that allows for efficient sampling and weighting representativeness. Our survey median completion time was 14.58 min, and the completion rate of 60 % yielded 1207 responses from across the US. The survey instrument was designed with a focus on issues of environmental health hazards and thus offers appropriate data to assess questions about public attitudes in this context.^5^

#### Dependent variable: protective behavior

We measure the dependent variable with respondents' answers to a question about actions taken facing potential exposure to different environmental hazards. Respondents marked the relevant actions from the following: (1) Drink bottled, filtered, or boiled water, (2) Wash fresh produce, (3) Eat certified organic foods, (4) Stay indoors when the outdoor air is smoggy or smoky, (5), Use specialized products like as air filters, bedding, or cleaning products, (6) Clean house or clothes more often. To quantify protective behavior, we first create a count variable for the number of actions taken (0 for no actions, 6 for all). Then, we transformed this count indicator into a proportional variable by dividing each respondenťs count by six, resulting in values ranging from 0 to 1, representing the extent to which individuals engaged in the various protective behaviors.

Our approach to test protective behavior is not unique [10,73] and most studies test similar type of actions or ask about pro-environmental behavior [30]. We analyze a broader and more diverse set of options that accounts for food consumption [26,31], purchasing or installing house cleaning products [19,31], and refraining from outdoor activities [17, 26]. By covering a wide range of actions that address different health threats, we provide more comprehensive results about the drivers of protective behavior.

#### Independent variables

##### Risk perception (Appraisal).

We assess risk perceptions based on responses to the question: “How concerned are you about being exposed to environmental and health threats from the following sources?” There are 11 items, representing various sources: (1) drinking water, (2) air, (3) food, (4) agriculture, (5) mining, (6) consumer products, (7) industrial sites, (8) abandoned polluted sites, (9) treated wastewater, (10) municipal solid waste disposal, (11) soil. Responses are measured on a 1–4 scale (1=Not concerned at all; 4= Very concerned). Based on the 11 items, we computed an index score (α=0.96) to measure individuals' health-related risk perceptions.

##### Awareness (Antecedent 1).

We measure respondents’ awareness of environmental and health issues based on the question, “How concerned are you about the following public issues?” We compute an index score (α=0.90) for four items: (1) pollution, (2) health care, (3) local environmental health threats, and (4) the environment.

##### Knowledge (Antecedent 2).

Scholars discuss two broad types of knowledge: perceived knowledge and factual (i.e., actual) knowledge. Perceived knowledge is a cognitive process that can determine individual action based on the form of competence and ability, while factual knowledge represents objective, verifiable information that may not always align with an individual's self-assessments. Perceived knowledge influences decision-making through confidence or subjective understanding, and factual knowledge provides a concrete foundation for informed actions, though its impact may vary depending on how individuals interpret or apply it in real-world situations [74].

We capture both types of knowledge with two separate indicators. First, *Perceived knowledge,* is an index score (α=0.92) based on the question: “How knowledgeable would you say you are about each of the following environmental threats to people’s health?” Answers include contaminations to drinking water or food, air pollution, pollution of solid waste and wastewater, and chemicals in consumer products. Responses are measured on a 1–4 scale (1=Not knowledgeable at all to 4=Very knowledgeable). Second, *Factual knowledge* is based on a series of “True” or “False” statements related to environmental hazards. We code each correct response as 1 and all incorrect or Don’t know responses as 0. Then, we tally the four items to create a score for each respondent.

##### Experience (Antecedent 3).

Individuals experience with environmental threats serves as a key antecedent influencing behavior and intentions to mitigate adverse impacts [5]. This variable is captured using a self-assessment approach similar to the procedure for rating self-reported health conditions [75]. We measure respondents’ experience of health threats with two variables. First, respondents rate the state of health in their household (ranging from 1=Excellent to 5=Poor). Second, we use a direct indicator (*Chronic health*) that is asking whether someone in respondents’ households has been diagnosed with a chronic health condition (dichotomous, “Yes” or “No”).

##### Perceived government competence (Coping appraisal).

Studies show that perceived competence in institutions (e.g., government) varies in its influence on individuals’ protective behavior [76]. High levels of perceived agencies’ competence may reduce individuals' engagement in protective behaviors, as they rely on institutional responses. Conversely, when trust and/or perceived competence is low, individuals may take proactive measures to protect themselves.

We measure respondents' views of government agencies’ competence to address environmental hazards. The survey item asks “How incompetent or competent do you think each of these agencies is at regulating sources of environmental health threats?”: (1) EPA (U.S. Environmental Protection Agency), (2) FDA (U.S. Food and Drug Administration), (3) Your state environmental quality agency, (4) Your state public health agency, (5) Your local public health agency. Respondents are measured on 1–5 scale (from 1=Very incompetent to 5=Very competent), and we computed an index score (α=0.88).

Lastly, we measure respondents’ socioeconomic characteristics: age, gender, education and income level.

#### Analytic strategies

We implement a two-stage empirical analysis of our survey data. First, we examine the relationships between the independent variables and our outcome variables by estimating several ordinary least squares (OLS) models which is the common practice and provides intuitively interpretable coefficients that facilitate comparison with prior research. The mean variance inflation factor for our model (VIF=1.27) indicate limited multicollinearity issues. Then, since our dependent variable is a proportional indicator ranging from 0 to 1, we employ Fractional logit regression models that are specifically designed for variables restricted to a proportional variable, ensuring predicted values remain within valid bounds and providing consistent estimates under general distributional assumptions [77]. With the fractional logistic regression, we estimate the conditional mean of the proportional dependent variable relative to the linear predictor [78].

Our proportional dependent variable is right-skewed but not highly zero-inflated. We implement fractional logistic regression while accounting for quasi-likelihood estimates, incorporating asymmetric and heteroscedastic variables in the analysis. In the results section, we present both sets of models – the OLS model serves as the benchmark while the fractional logit estimates are a robustness test for the relationship, and offer a better fit considering the distribution of the outcome variable.

In the second step of the empirical analysis, we use structural equation models (SEM) to investigate how the risk and coping appraisals operate as mediators that transmit the effects of the various antecedents onto the dependent variable of protective actions [5,46]. We employ two mediators: risk perception and government competence which transmit the effects of the antecedent factors (i.e., knowledge, awareness, etc.) onto protective actions (the outcome variable). In addition, the mediators can also have direct effect on the outcome. Risk perception can enhance individuals’ protective intentions while views about high levels of competence of the government can lower individuals’ protective intention [18,79]. Therefore, we test the direct as well as mediating roles of these two variables. Finally, the SEM approach also offers the benefit of examining the structural relationships linking the different factors (variables) that we test as drivers of risk, views of competence and protective behavior [8].^6^

Overall, the analytic design follows directly from the logic of PMT. The first-stage OLS and fractional-logit models estimate the direct associations between threat and coping appraisals and protective behavior, corresponding to PMT’s proposition that these appraisals jointly determine behavioral motivation [1]. The second-stage SEM analysis operationalizes the causal sequence proposed by PMT as we test how antecedent factors (awareness, knowledge, and experience) influence protective behavior through their effects on risk and coping appraisals. This two-step approach allows us to assess both direct and mediated pathways consistent with the theoretical structure of PMT and provides a statistically coherent translation of its behavioral logic.

## Results

We begin the analysis with a series of tests in which we estimate the relationships between the independent variables and the outcome of protective behavior using OLS and fractional logit regression models. Table 1 presents these models.

The results of Model 1–1 display positive and significant coefficients for risk perception (β=0.4, *p* < 0.01), perceived knowledge (β=0.14, *p* < 0.0001), factual knowledge (β=0.327, *p* < 0.001), and chronic disease (β=0.509, *p* < 0.01). We also find that health experience is negatively associated with respondents’ mitigation behaviors (β= − 0.024, *p* < 0.001). Therefore, those who display higher risk perceptions and knowledge of the hazards engage in larger proportion of protective actions. On the other hand, those facing worsening health conditions in their household partake in less actions. While the overall explained variance of our model is modest (R^2^ = 0.19), this level is consistent with prior empirical studies applying PMT and related behavioral frameworks. Behavioral models predicting protective or pro-environmental actions rarely achieve high explained variance because these behaviors are shaped by multifaceted social, emotional, and contextual influences that are difficult to capture empirically. For example, Bubeck et al. [28] reported similar levels of explained variance when examining flood-coping behavior. Studies modeling pro-environmental behavior also document R^2^ values in the range of 0.10–0.25 [30,46]. Within PMT, low-to-moderate R^2^ values are theoretically expected because the framework emphasizes cognitive appraisals of risk and coping evaluations as psychological mechanisms that motivate behavior rather than deterministic predictors. Hence, our modest R^2^ reflects the complexity of human decision-making facing environmental health risk and should be interpreted as theoretically meaningful rather than statistically weak.

Considering the nature of our outcome variable, we conduct a robustness test using a fractional logit regression model which offers better estimates for proportional variable. The results in Model 1–2 are consistent with Model 1–1, increasing our confidence in the strength of the results. Specifically, we find that a one-unit increase in perceived risk is associated with a 26 % increase in the odds of adopting a greater proportion of protective actions. The coefficient for perceived knowledge is even stronger indicating that a one-unit increase corresponds to almost 1.5 times the likelihood of adopting protective behaviors, while a one-unit increase in factual knowledge is associated with a more modest 23 % increase. Regarding health factors, worsening household health is linked to a 13 % decrease in the odds of adopting protective behaviors, whereas households with chronic illness exhibit a 36 % increase in the odds of engaging in such actions.

In the conceptual framework, we posit that both threat (i.e., risk perception) and coping (i.e., government competence) appraisals serve as mediating variables. To test this proposition, we run a mediation analysis to explore how the antecedent individual-level factors (i.e., awareness, knowledge, and experience) are associated with the threat and coping appraisals. To support the mediating relationship, three conditions must be met: (1) the independent variables are related to a dependent variable, (2) the independent variables are related to the mediating variables, and (3) the mediating variables are related to a dependent variable [80]. The results of Table 1 satisfy the first condition. In Table 2, we use OLS regression models to test the second and third conditions.

Models 2–1 and 2–2 test the effects of all antecedents on the two mediating variables (i.e., threat and coping appraisal). The awareness factor is positive for both models (Model 2–1 / β= 0.526, *p* < 0.001; Model 2–2 / β= 0.284, *p* < 0.001) suggesting that individuals who are more aware of environmental health issues display higher risk perceptions and view the government as competent in addressing these threats.

We also find that respondents’ perceived knowledge is positively associated with risk perception (Model 2–1 / β=0.254, *p* < 0.001) while it is negatively associated with government competence (Model 2–2 / β= − 0.118, *p* < 0.01). On the other hand, respondents’ factual knowledge shows the inverse effects with negative association with risk perception (Model 2–1 / β= − 0.054, *p* < 0.01) and positive with government competence (Model 2–2 / β=0.055, *p* < 0.05). The health experience factor displays a positive association with increased risk perception (Model 2–1 / β= 0.060, *p* < 0.01) and negative with government competence (Model 2–2 / β= − 0.117, *p* < 0.001). The chronic health factor fails to reach statistical significance. These results offer evidence to satisfy condition 2. In addition, we find some contrasting relationships with both mediators.

In models 2–3 and 2–4, we assess condition 3 for mediation by testing how the mediating factors relate to the dependent variable while controlling for all independent variables. We find a positive association of the risk perceptions mediator (Model 2–3 / β= 0.04, *p* < 0.01) with engaging in higher proportion of protective actions. Therefore, respondents who report increased risk perceptions tend to engage in higher proportion of protective behavior. On the other hand, the coping appraisal coefficient is not significant indicating that views of the government’s role are not associated with protective behavior.

**Fig. 1.**
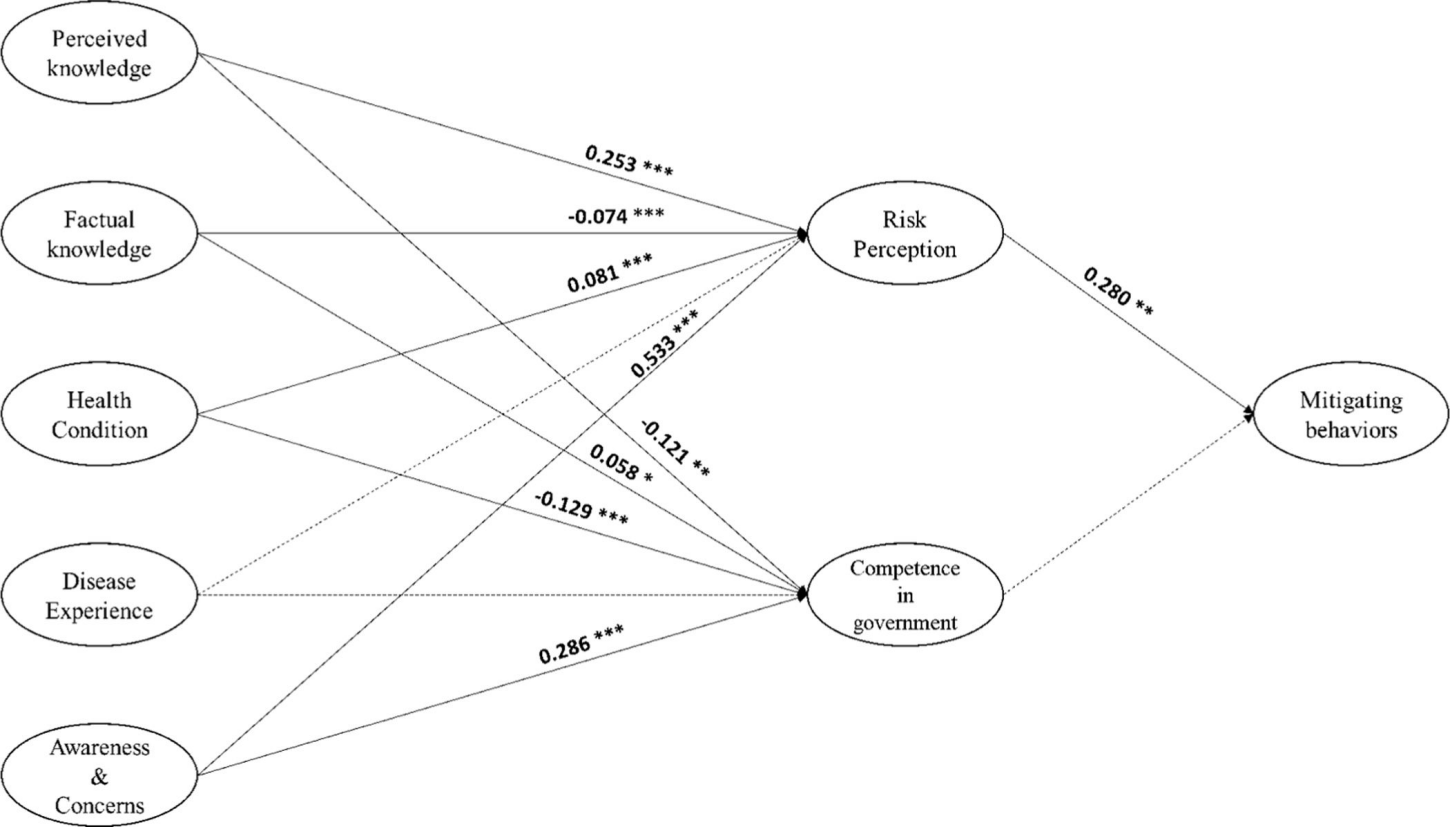
Structural model and mediating factors – direct effects.

The results of the models in Table 2 suggest that our indicators of threat and coping appraisals serve as mediators when assessing the drivers of protective behavior. In a second set of tests, we implement structural equation modeling (SEM) to assess the structural relationship of our variables and in particular, how the threat and coping appraisals operate as mediators and affect the protective behaviors. For our SEM test, multiple metrics suggest a good overall model fit (χ^2^(9) = 31.77(*p* < 0.001), RMSEA = 0.046, CFI = 0.977, TLI = 0.923, SRMR = 0.016). In Fig. 1, we plot the results of the SEM and the direct effects of the various factors on the mediating variables and the outcome of protective behavior.

The results of the SEM analysis correspond with the both sets of regression models. First, we find that perceived knowledge, health experience, and awareness are positively associated with risk perception while factual knowledge is negatively associated with risk perception. On the other hand, perceived knowledge and health experience are negatively associated with government competence while factual knowledge and awareness are positively associated with government competence. The path analysis demonstrates the direct effect of risk perceptions on protective behavior while competence views are not directly related to protective actions.

In Table 3, we present a more comprehensive picture of the analysis by detailing the direct and indirect effects of all variables on the dependent variable of protective behavior. Other than re-affirming the relationship of both risk perception and government competence with behaviors, the results provide evidence on the indirect and direct effects of some of the antecedent factors on the mediators as well as the outcome variable. Some of the effects presented in Table 3 demonstrate substantive as well as statistical importance. The risk perception predictor is meaningful as higher perceptions consistently translate into increased engagement in protective behaviors (total effect = 0.28). Likewise, perceived knowledge exerts a strong practical effect (total effect = 1.062), while factual knowledge is more modest. These effect sizes indicate that enhancing public risk awareness and knowledge can directly increase protective behavior. At the same time, increased awareness and knowledge also indirectly motivate mitigation by enhancing risk perception which motivate protective behaviors.

## Discussion

In this study, we investigate the determinants of citizens' decision to engage in health-related protective behavior in response to environmental health threats. Our work focuses on hazards that are relatively under-explored such as water and soil contamination, air pollution and toxicants spillage. We build on protection motivation theory (PMT) [1, 27] and argue that the decision to act depends on risk perceptions (threat appraisal) as well as individuals’ views of the role of local authorities in addressing these health risks (coping appraisal). Using a national sample of Americans, we find support for the role of risk perceptions (threat appraisal). At the same time, the coping appraisal (government role) does not seem to be associated with protective behavior.

PMT stipulates that threat appraisal plays an important role in driving mitigation. We find substantial evidence that support a direct and powerful association between risk perceptions and engaging in different actions to protect one’s health facing pollution-type hazards. In our empirical analysis, using both regression and SEM approaches we find that risk perceptions consistently display a positive relationship with engaging in a larger proportion of protective actions. This finding fits with most studies that examine mitigation of natural hazards [38–40]. More specifically, we add to a recent review of PMT studies and demonstrate that risk perceptions are essential for behavior [30].

The role of risk perceptions is also a central finding to studies that explore mitigation of pollution or contamination-type hazards [43,44, 81]. Little et al. [31] use PMT to address health-related behavior facing water pollution. In their survey results, threat appraisal was more essential to health-focused protective behavior compared to actions to protect the environment. This further bolster our theoretical argument and empirical findings that facing health threats from pollution-type hazards, risk perceptions are central to the decision to act.

While we find substantial support for the role of risk perceptions, our coping appraisal factor of government competence is not associated with protective actions. One of our main arguments is that individuals place more emphasis on centralized (government) response to pollution-type hazards as those require more expertise, similar to addressing health risks like the global pandemics [79,82]. Our null results suggest that government efficacy is less important in this context, and citizens rely more on personal risk perceptions and efficacy, a result documented by other studies of mitigation of environmental health hazards [31,83]. Beyond that, these findings offer a potential contextual refinement of PMT, indicating that coping appraisal mechanisms may function differently across hazard types. It seems that in chronic, diffuse, and human-made risks such as pollution or contamination individuals are more likely to internalize coping responsibility, and place greater emphasis on personal efficacy rather than institutional trust. This point captures some of the conclusions offered by Kothe et al. [30] about the need for additional work on the definition and measures of PMT’s main constructs. Our results suggest that PMT’s explanatory scope, especially the coping appraisal, may need to balance between institutional and individual efficacy and how it may be adjusted based on the characteristics of various hazard contexts.

Irrespective of specific measurement or definition issues, our findings, and in particular the lack of effect for government’s role in shaping protective behavior should serve as a warning sign to local authorities and governments as a whole. In the face of health risks from pollution and contamination-type hazards, when citizens contemplate how to protect themselves, the potential role of governments in enhancing community resilience does not seem to play a part in their calculus, and they view it as “it’s up to us” to prevent the negative health outcomes of environmental hazards. One possible way to address this issue is for public healthcare agencies and local environmental organizations to invest in helping residents, especially of at-risk communities, to learn more about, and manage the potential health risks of pollution-type hazards. Solutions may include implementing communication strategies within community workshops or education campaigns emphasizing how the government can help and provide information on direct health risks and protection behavior. Such campaigns also offer opportunities for institutional collaboration between local health centers with schools and other community organizations that spread the messages on recommended mitigation actions. If our results show that citizens view the government as “irrelevant”, then “street-level” agencies and groups should take the lead in strengthening community engagement and foster stronger connections with residents to help them be better prepared in case of disasters.

In addition, our results display rather modest R^2^ values across the models. These findings are defensible in the present context because the dependent variable represents voluntary protective behavior driven by multiple unobserved forces including emotions or situational exposure. Behavioral models grounded in social-psychological theory often explain a relatively small share of total variance yet remain substantively powerful in identifying the direction and mechanism of effects [14, 46]. As these studies emphasize, the goal is not maximal prediction but theoretical coherence, i.e. tracing how antecedent factors shape threat and coping appraisals that in turn influence motivation to act. Thus, our findings align with established empirical patterns in environmental risk and health-behavior research, reinforcing the theoretical validity of PMT despite modest statistical magnitude.

Our analysis also demonstrates the mediating role of risk perception in shaping behavior. In particular, we find how several antecedent factors shape risk perceptions, which in turn, have a direct effect on adopting protective behavior. These findings correspond with studies that model risk perceptions as part of a sequential process ending with adopting protective behavior [10,30,32,34].

Using both regression and SEM, we employ a mediating approach and demonstrate the direct effects of antecedent factors such as awareness, knowledge and experience of health issues on risk perceptions and government competence, as well as their indirect effects on engaging in protective behavior.

Awareness of the hazards is an important antecedent factor. We find that it has a direct effect on risk perceptions, which fit with other studies of health-specific risk perceptions [50,58]. In addition, awareness has an indirect effect on behavior. In a similar fashion, Bradley et al. [46] model risk perception as mediator for the affect factor (like our awareness) and show its indirect effect on pro-environmental behavior. These results have clear practical importance as investing in increasing public awareness can enhance risk attitudes and in turn, public resilience by adopting protective actions in response to environmental hazards.

We measure knowledge using both perceived and factual understanding of the hazards. While perceived knowledge is akin to familiarity, factual knowledge relies on legitimate and scientific information [84]. Overall, both measures display the expected effects on behavior in the context of a mediating model. We find that both perceived and factual knowledge have a positive total effect on behavior, a result that is prevalent across studies of climate change and natural hazards resilience [46,55,56]. At the same time, we find that factual knowledge has a negative association with risk perception and a negative indirect effect on behavior. With respect to risk perceptions, this may point to increased sense of control and thus reduced risk assessments [23]. Considering behavior, this is an interesting finding. Little et al. [31] find that knowledge is associated with behavior to protect the environment, but not actions to protect one’s health. Our negative findings may suggest that individuals with clearer understanding of the threats know how to protect themselves, and thus only engage in mitigating activities that they think are effective.

## Conclusion

This study explores the factors that shape protective behavior facing less common environmental hazards. Using PMT [1,2] we study how threat and coping appraisals drive the decision to engage in several actions to reduce health risks from hazards. Our analysis of public survey of Americans offers insights on risk assessments of such threats, and how views about the role of authorities can help or harm protection.

Our work offers several interesting additions to the literature. First, the majority of studies exploring pollution-type threats tend to assess one or two types of hazards [19,26,31,41]. We offer a broader assessment by exploring multiple types of hazards which can result from natural disasters or due to man-made processes. Second, we extend research on protective behavior by analyzing diverse types of actions related to consumption, cleaning or limiting outdoor activities. By analyzing multiple hazards and types of actions we conduct a more comprehensive test and offer multiple interesting insights. Third, we implement PMT in the underexplored issue area of pollution-type hazards and reconceptualize the coping appraisal as views of government competence or capacity to address the threat. Considering the central role of authorities in mitigating such hazards, our null findings suggest the need to clarify how views of government competence matter for the coping appraisal. By elucidating the role of the government, alongside self-efficacy, as two dimensions of coping, researchers can extend PMT and use its theoretical expectations to explain protective behavior facing environmental hazards that receive less scholarly attention. Fourth, most studies employ a single method (regression or SEM) in their empirical testing stage. We implement a multi-method approach that tests the structural relationship and complex inter-linkages between multiple antecedent factors (awareness, knowledge, etc.), mediators (risk perceptions and government competence) and the observed outcome of protective actions.

Despite the encouraging results, our work is still limited in several aspects and warrants further research. First, we conceptualize coping appraisal as views of government competence to address threats. The null effects of this factor suggest protective behavior is driven mostly by self-efficacy. Yet, employing more specific measures of government competence (such as views of particular solutions) may better capture this concept. Another possibility that future work should explore is to measure both self-efficacy and government efficacy as two dimensions of coping appraisal. As such, our results also provide an opportunity to refine the theoretical boundaries of PMT. The non-significant role of government competence underscores that coping appraisal may vary by hazard type. Studies that explicitly account for the contextual locus of efficacy (separate institutional and individual coping orientations) can better captures the dynamics of protective behavior across diverse environmental hazards. Second, our data (representative sample of US citizens) allows us to offer general insights about behavior facing environmental pollution. Yet, collecting cross-national samples or testing this framework on more vulnerable populations can provide additional insights about the broader application of PMT as well as more specific lessons for protective behavior of at-risk population segments. Third, our measure for behavior is limited as it only captures the intentions aspect, and intentions do not always lead to behavior [30]. Potential solutions for the issue are complex and require additional resources (conducting follow-ups on actual behavior of individuals facing pollution hazards) or applying experimental tools that can measure behavior more accurately.

This study underscores the crucial role of risk perceptions and institutional trust/competence in motivating protective health behavior in the face of less common environmental hazards like pollution and contamination. Further analysis of what drives mitigating behavior can provide more insights about the factors that encourage protective actions in the face of health hazards that are becoming more prevalent in the contemporary world.

## Figures and Tables

**Table 1:** The drivers of protective behavior – OLS & Fractional Logit Regression models.

DV: Mitigating behavior	Model 1–1 OLS	Model 1–2 Fractional Logit
Risk perception	0.0400**	0.234**
	*(0.0133)*	*(0.0804)*
Government Competence	−0.00973	−0.0617
	*(0.00977)*	*(0.0561)*
Awareness	0.0167	0.108
	*(0.0127)*	*(0.0724)*
Perceived knowledge	0.140***	0.882***
	*(0.0144)*	*(0.0947)*
Factual knowledge	0.0327***	0.204***
	*(0.00817)*	*(0.0475)*
Health Experience	−0.0240*	−0.140*
	*(0.00995)*	*(0.0579)*
Chronic Health	0.0509**	0.310**
	*(0.0169)*	*(0.0943)*
Age	−0.000622	−0.00348
	*(0.000450)*	*(0.00277)*
Gender	−0.00395	−0.0161
	*(0.0156)*	*(0.0879)*
Education	0.0471***	0.303***
	*(0.00853)*	*(0.0519)*
Income	0.00445	0.0263
	*(0.00505)*	*(0.0304)*
Constant	−0.344***	−4.999***
	*(0.0712)*	*(0.440)*
Observations	1177	1177
R-squared	0.190	
Wald chi-square		256.62

Note:

*** *p* < 0.001

** *p* < 0.01

* *p* < 0.05.

**Table 2:** Mediation analysis – OLS regression models.

Dependent Variables	Risk perception	Government Competence	Protective behaviors	Protective behaviors
	Model 2–1	Model 2–2	Model 2–3	Model 2–4
Risk perception			0.0405**	
			*(0.0133)*	
Government Competence				−0.0108
				*(0.00979)*
Awareness	0.526***	0.284***	0.0137	0.0380***
	*(0.0223)*	*(0.0303)*	*(0.0123)*	*(0.0105)*
Perceived knowledge	0.254***	−0.118**	0.141***	0.150***
	*(0.0308)*	*(0.0420)*	*(0.0144)*	*(0.0141)*
Factual knowledge	−0.0541**	0.0554*	0.0322***	0.0306***
	*(0.0179)*	*(0.0244)*	*(0.00816)*	*(0.00817)*
Health experience	0.0603**	−0.117***	−0.0228*	−0.0217*
	*(0.0217)*	*(0.0296)*	*(0.00989)*	*(0.00996)*
Chronic Health	−0.00648	0.0142	0.0508**	0.0507**
	*(0.0371)*	*(0.0506)*	*(0.0169)*	*(0.0169)*
Age	0.00138	−6.78e-05	−0.000622	−0.000567
	*(0.000991)*	*(0.00135)*	*(0.000450)*	*(0.000451)*
Gender	0.0487	0.0513	−0.00448	−0.00195
	*(0.0342)*	*(0.0466)*	*(0.0155)*	*(0.0156)*
Education	−0.0659***	0.00740	0.0471***	0.0445***
	*(0.0187)*	*(0.0255)*	*(0.00853)*	*(0.00852)*
Income	−0.0182	0.0194	0.00427	0.00374
	*(0.0111)*	*(0.0151)*	*(0.00504)*	*(0.00506)*
Constant	0.514***	2.718***	−0.371***	−0.320***
	*(0.145)*	*(0.197)*	*(0.0660)*	*(0.0710)*
Observations	1177	1177	1177	1177
R-squared	0.424	0.092	0.189	0.183
Wald chi-square				

Note:

*** *p* < 0.001

** *p* < 0.01

* *p* < 0.05.

**Table 3:** Direct, indirect, and total effects of SEM analysis.

Predictor	Direct Effects			Indirect Effects	Total Effects
	Risk perception	Competence	Mitigating behaviors	Mitigating behaviors	Mitigating behaviors
Risk perception			0.280**		0.280**
Competence			−0.068		−0.068
Awareness	0.533***	0.286***	0.117	0.130*	0.246***
Perceived knowledge	0.252***	−0.121**	0.983***	0.079**	1.062***
Factual knowledge	−0.074***	0.058*	0.229***	−0.025**	0.204***
Health experience	0.081***	−0.130***	−0.168*	0.032*	−0.136*
Chronic Health	0.001	0.018	0.356**	−0.001	0.355**
Age			−0.004		−0.004
Gender			−0.028		−0.028
Education			0.330***		0.330***
Income			0.031		0.031

Note:

*** *p* < 0.001

** *p* < 0.01

* *p* < 0.05.

## Footnotes

1 NPR Link

2 USA Today Link

3 Some distinguish between mitigation and adaptation [85]. This distinction is beyond the scope of this study and we use the terms mitigation, adaptation, and responses interchangeably.

4 Response and self-efficacy also shape protective actions in studies that explore pollution-type hazards, yet do not rely on PMT [17,19].

5 Survey instrument approved by Texas A&M IRB (ID: IRB2021–0430M).

6 We employ multiple tests to assess the validity of the indicators and the method. We test construct validity by calculating AVE (average variance extracted) for each construct. The AVE for the variables indicates that a good amount of variance (50–80%) is captured by the factors. We test convergent validity by estimating the factor loading from all items (all are significant). In addition, the range of Cronbach’s alpha values (0.88 – 0.96) for all constructs suggest that the indicator items for each construct correlate with each other to an adequate degree and that convergent validity is established for all the constructs. Tests of discriminant validity show that all correlation coefficients among constructs are below the 0.85 standard (Fornell & Larcker, 1981). Lastly, to alleviate concerns of common method bias, we conducted Harman’s single factor test. The results indicate that total variance extracted by one single factor (31.36%) is below the 50% threshold, suggesting that common method bias is not a concern. A detailed view of the results of all tests is available in Table 1A in the appendix.

## References

[1] E Maddux J, W Rogers R, Protection motivation and self-efficacy: a revised theory of fear appeals and attitude change, J. Exp. Soc. Psychol. 19 (5) (1983) 469–479, 10.1016/0022-1031(83)90023-9.

[2] Rogers RW, A protection motivation theory of fear appeals and attitude Change1, J. Psychol. 91 (1) (1975) 93–114, 10.1080/00223980.1975.9915803. 28136248

[3] Dvir R, Goldsmith C, Seavey I, Vedlitz A, Local-level managers’ Attitudes towards natural hazards resilience: the case of Texas, Environ. Pollut. November (2022) 1–21, 10.1080/17477891.2022.2141178.

[4] Houser M, Gazley B, Reynolds H, G Browning E, Sandweiss E, Shanahan J, Public support for local adaptation policy: the role of social-psychological factors, perceived climatic stimuli, and social structural characteristics, Glob. Environ. Change 72 (January) (2022) 102424, 10.1016/j.gloenvcha.2021.102424.

[5] Hwang H, Vedlitz A, Bixler RP, Growing community resilience from the grassroots: risk awareness, confidence in institutions, and civic participation in a natural hazards context, Nat. Hazards. Rev. 24 (3) (2023) 04023020, 10.1061/NHREFO.NHENG-1658.

[6] Portney KE, Hannibal B, Goldsmith C, McGee P, Liu X, Vedlitz A, Awareness of the food–Energy–Water nexus and public policy support in the United States: public attitudes among the American people, Environ. Behav. 50 (4) (2018) 375–400, 10.1177/0013916517706531.

[7] Singh AS, Zwickle A, Bruskotter JT, Wilson R, The perceived psychological distance of climate change impacts and its influence on support for adaptation policy, Environ. Sci. Policy. 73 (July) (2017) 93–99, 10.1016/j.envsci.2017.04.011.

[8] Jadil Y, Ouzir M, Exploring the predictors of health-protective behavior during the COVID-19 pandemic: a multi-country comparison, Environ. Res. 199 (2021) 111376, 10.1016/j.envres.2021.111376. PMC9750228 34043969

[9] (edited by Skinner C, Tiro J, Champion V, The health belief model, in: Rimer V, Glanz(Eds.), Health Behavior: Theory, Research, and Practice, Wiley, Germany, 2015, pp. 75–94 (edited by.

[10] Batool N, Wani MD, Shah SA, Dada ZA, Theory of planned behavior and value-belief norm theory as antecedents of pro-environmental behaviour: evidence from the local community, J. Hum. Behav. Soc. Environ. 34 (5) (2023) 693–709, 10.1080/10911359.2023.2205912.

[11] Kollmuss A, Agyeman J, Mind the gap: why do people act environmentally and what are the barriers to pro-environmental behavior? Environ. Educ. Res. 8 (3) (2002) 239–260, 10.1080/13504620220145401.

[12] Li D, Zhao L, Ma S, Shao S, Zhang L, What influences an individual’s pro-environmental behavior? A literature review, Resour. Conserv. Recycl. 146 (July) (2019) 28–34, 10.1016/j.resconrec.2019.03.024.

[13] Stern PC, New environmental theories: toward a coherent theory of environmentally significant behavior, Journal of Social Issues 56 (3) (2000) 407–424, 10.1111/0022-4537.00175.

[14] Van Valkengoed AM, Steg L, Meta-analyses of factors motivating climate change adaptation behaviour, Nat. Clim. Chang. 9 (2) (2019) 158–163, 10.1038/s41558-018-0371-y.

[15] Kievik M, Gutteling JM, Yes, we can: motivate Dutch citizens to engage in self-protective behavior with regard to flood risks, Nat. Hazards. 59 (3) (2011) 1475–1490, 10.1007/s11069-011-9845-1.

[16] Zhao C, Zhang M, Wang W, Exploring the influence of severe haze pollution on residents’ Intention to purchase energy-saving appliances, J. Clean. Prod. 212 (March) (2019) 1536–1543, 10.1016/j.jclepro.2018.12.134.

[17] Lin TTC, Bautista JR, Predicting intention to take protective measures during haze: the roles of efficacy, threat, Media trust, and affective attitude, J. Health Commun. 21 (7) (2016) 790–799, 10.1080/10810730.2016.1157657. 27315440

[18] Tan H, Xu J, Differentiated effects of risk perception and causal attribution on public behavioral responses to air pollution: a segmentation analysis, J. Environ. Psychol. 65 (2019) 101335, 10.1016/j.jenvp.2019.101335.

[19] Wei J, Zhu W, Marinova D, Wang F, Household adoption of smog protective behavior: a comparison between two Chinese cities, J. Risk. Res. 20 (7) (2016) 846–867, 10.1080/13669877.2015.1121904.

[20] Davison SMC, White MP, Pahl S, Taylor T, Fielding K, Roberts BR, Economou T, McMeel O, Kellett P, Fleming LE, Public concern about, and desire for research into, the Human health effects of marine plastic pollution: results from a 15-country survey across Europe and Australia, Glob. Environ. Change 69 (July) (2021) 102309, 10.1016/j.gloenvcha.2021.102309.

[21] Potts T, Pita C, O’Higgins T, Mee L, Who cares? European attitudes towards marine and coastal environments, Mar. Policy. 72 (October) (2016) 59–66, 10.1016/j.marpol.2016.06.012.

[22] Kramm J, Steinhoff S, Werschmöller S, Völker B, Völker C, Explaining risk perception of microplastics: results from a representative survey in Germany, Glob. Environ. Change 73 (March) (2022) 102485, 10.1016/j.gloenvcha.2022.102485.

[23] Deng L, u Cai L, Sun F, Li G, Che Y, Public Attitudes towards microplastics: perceptions, behaviors and policy implications, Resour. Conserv. Recycl. 163 (December) (2020) 105096, 10.1016/j.resconrec.2020.105096.

[24] S Jung Y, Sampath V, Prunicki M, Aguilera J, Allen H, LaBeaud D, Veidis E, , Characterization and regulation of microplastic pollution for protecting planetary and Human health, Environ. Pollut. 315 (December) (2022) 120442, 10.1016/j.envpol.2022.120442. PMC12077371 36272609

[25] Kim G, Kim S, Hwang E, Searching for evidence-based public policy and practice: analysis of the determinants of personal/public adaptation and mitigation behavior against particulate matter by focusing on the roles of risk perception, communication, and attribution factors, Int. J. Environ. Res. Public Health 18 (2) (2021) 428, 10.3390/ijerph18020428. 33430400 PMC7827748

[26] Ross AD, Hotard A, Kamalanathan M, Nolen R, Hala D, Clay LA, Quigg A, Awareness is not enough: frequent use of water pollution information and changes to risky behavior, Sustainability. 12 (20) (2020) 8695, 10.3390/su12208695.

[27] Mertens K, Jacobs L, Maes J, Poesen J, Kervyn M, Vranken L, Disaster risk reduction among households exposed to landslide hazard: a crucial role for self-efficacy? Land. use policy. 75 (June) (2018) 77–91, 10.1016/j.landusepol.2018.01.028.

[28] Bubeck P, Wouter Botzen WJ, Laudan J, Aerts JCJH, Thieken AH, Insights into flood-coping appraisals of protection motivation theory: empirical evidence from Germany and France: insights into flood-coping appraisals of protection motivation theory, Risk Anal. 38 (6) (2018) 1239–1257, 10.1111/risa.12938. 29148082

[29] Hu SY, Yu M, Que T, Fan G, Xing HG, Individual willingness to prepare for disasters in a geological hazard risk area: an empirical study based on the protection motivation theory, Nat. Hazards. (2022) 1–25, 10.1007/s11069-021-05026-8.

[30] Kothe EJ, Ling M, North M, Klas A, Mullan BA, Novoradovskaya L, Protection motivation theory and pro-environmental behaviour: a systematic mapping review, Aust. J. Psychol. 71 (4) (2019) 411–432, 10.1111/ajpy.12271.

[31] Little GM, Kohl PA, Wardropper CB, Health and environmental protective behavioral intentions for reducing harm from water pollutants, Environ. Manage (2023) 1–11, 10.1007/s00267-023-01805-0. PMC9984752 36869914

[32] Bhandari G, Lalit BC, Sapkota U, , Safety Behavior of Nepalese Strawberry Farmers as Reflected by the Protection Motivation Theory, Int. J. Environ. Res. 19 (2025) 71, 10.1007/s41742-024-00726-y.

[33] Slovic P, Fischhoff B, Lichtenstein S, Why study risk perception? Risk Anal. 2 (2) (1982) 83–93, 10.1111/j.1539-6924.1982.tb01369.x.

[34] Wachinger G, Renn O, Begg C, Kuhlicke C, The risk perception paradox-implications for governance and communication of natural hazards: the risk perception paradox, Risk Anal. 33 (6) (2013) 1049–1065, 10.1111/j.1539-6924.2012.01942.x. 23278120

[35] Spence A, Poortinga W, Pidgeon N, The psychological distance of climate change: psychological distance of climate change, Risk Anal. 32 (6) (2012) 957–972, 10.1111/j.1539-6924.2011.01695.x. 21992607

[36] Xie B, Brewer MB, Hayes BK, McDonald RI, Newell BR, Predicting climate change risk perception and willingness to act, J. Environ. Psychol. 65 (October) (2019) 101331, 10.1016/j.jenvp.2019.101331.

[37] Linden S, The social-psychological determinants of climate change risk perceptions: towards a comprehensive model, J. Environ. Psychol. 41 (March) (2015) 112–124, 10.1016/j.jenvp.2014.11.012.

[38] Antronico L, De Pascale F, Coscarelli R, Gullà G, Landslide risk perception, social vulnerability and community resilience: the case study of Maierato (Calabria, Southern Italy), Int. J. Disaster Risk Reduct. 46 (June) (2020) 101529, 10.1016/j.ijdrr.2020.101529.

[39] Arbuckle JG, W Morton L, Hobbs J, Understanding farmer perspectives on Climate change adaptation and mitigation: the roles of trust in sources of climate information, climate change beliefs, and perceived risk, Environ. Behav. 47 (2) (2015) 205–234, 10.1177/0013916513503832. 25983336 PMC4359208

[40] Huang H, Huang J, Liu D, He Z, Understanding the public responses to landslide countermeasures in Southwest China, Int. J. Disaster Risk Reduct. 64 (October) (2021) 102500, 10.1016/j.ijdrr.2021.102500.

[41] Zhou Z, Liu J, Zeng H, Zhang T, Chen X, How does soil pollution risk perception affect farmers’ Pro-environmental behavior? The role of income level, J. Environ. Manage 270 (September) (2020) 110806, 10.1016/j.jenvman.2020.110806. 32507737

[42] Janmaimool P, Application of Protection Motivation Theory to Investigate Sustainable Waste Management Behaviors, Sustainability 9 (7) (2017) 1079, 10.3390/su9071079.

[43] M Heidbreder L, Bablok I, Drews S, Menzel C, Tackling the plastic problem: a review on perceptions, behaviors, and interventions, Science of The Total Environment 668 (June) (2019) 1077–1093, 10.1016/j.scitotenv.2019.02.437. 31018449

[44] Wu Y, Mo D, Liu J, Li Z, Chen X, Xie L, Public perception of microplastics on a popular Chinese social media platform, J. Clean. Prod. (2023) 137688, 10.1016/j.jclepro.2023.137688.

[45] Cooper CM, Langman JB, Sarathchandra D, Vella CA, Wardropper CB, Perceived risk and intentions to practice health protective behaviors in a mining-impacted region, Int. J. Environ. Res. Public Health 17 (21) (2020) 7916, 10.3390/ijerph17217916. 33126668 PMC7672644

[46] Bradley GL, Babutsidze Z, Chai A, Reser JP, The role of climate change risk perception, response efficacy, and psychological adaptation in pro-environmental behavior: a two nation study, J. Environ. Psychol. 68 (April) (2020) 101410, 10.1016/j.jenvp.2020.101410.

[47] Calculli C, D'Uggento AM, Labarile A, Ribecco N, Evaluating people's awareness about climate changes and environmental issues: a case study, J. Clean. Prod. 324 (2021) 129244, 10.1016/j.jclepro.2021.129244.

[48] M Lee T, Markowitz EM, Howe PD, Ko C-Y, Leiserowitz AA, Predictors of public climate change awareness and risk perception around the world, Nat. Clim. Chang. 5 (11) (2015) 1014–1020, 10.1038/nclimate2728.

[49] Poortinga W, Whitmarsh L, Steg L, Böhm G, Fisher S, Climate change perceptions and their individual-level determinants: a Cross-European analysis, Glob. Environ. Change 55 (March) (2019) 25–35, 10.1016/j.gloenvcha.2019.01.007.

[50] Akerlof K, Delamater P, Boules C, Upperman C, Mitchell C, Vulnerable populations perceive their health as at risk from climate change, Int. J. Environ. Res. Public Health 12 (12) (2015) 15419–15433, 10.3390/ijerph121214994. 26690184 PMC4690930

[51] Thaker J, Richardson LM, Holmes DC, Australians’ Perceptions about health risks associated with climate change: exploring the role of Media in a comprehensive climate change risk perception model, J. Environ. Psychol. 89 (August) (2023) 102064, 10.1016/j.jenvp.2023.102064.

[52] Catarino AI, Kramm J, Völker C, Henry TB, Everaert G, Risk posed by microplastics: scientific evidence and public perception, Curr. Opin. Green. Sustain. Chem. 29 (June) (2021) 100467, 10.1016/j.cogsc.2021.100467.

[53] Dvir R, Vedlitz A, The dark side of close-ties communities: how strong social connections shape health-related risk perceptions, Environ. Pollut. (2024) 1–20, 10.1080/17477891.2024.2445002. PMC12373392 40881647

[54] Begum M, Masud MM, Alam L, Mokhtar MB, Amir AA, The adaptation behaviour of marine fishermen towards climate change and food security: an application of the theory of planned behaviour and health belief model, Sustainability 14 (21) (2022) 14001.

[55] W Lim H, Fang D, Role of hazard information in the adoption of seismic hazard adjustments: information treatment experiment in Beijing, Int. J. Disaster Risk Reduct. 79 (September) (2022) 103182, 10.1016/j.ijdrr.2022.103182.

[56] Saphores J-DM, Ogunseitan OA, Shapiro AA, Willingness to engage in a pro-environmental behavior: an analysis of e-waste recycling based on a national survey of U.S. Households, Resour. Conserv. Recycl. 60 (March) (2012) 49–63, 10.1016/j.resconrec.2011.12.003.

[57] Dvir R, Vedlitz A, Mostafavi A, Far from home: Infrastructure, access to essential services, and risk perceptions about hazard weather events, Internat. J. Disas. Risk Reduc. 80 (2022) 103185, 10.1016/j.ijdrr.2022.103185.

[58] Dvir R, Vedlitz A, Ye X, Worried (and) sick: how environmental hazards affect americans’ health-related risk attitudes, Urban. Inform 3 (1) (2024) 26, 10.1007/s44212-024-00057-5.

[59] Hwang H, Bixler RP, Brown WA, Vedlitz A, How to activate nonprofit beneficiaries for community resilience? Examining the role of risk perception and evaluation of nonprofit services on prosocial behavior in the context of natural hazards, Sociol. Spectr. 44 (1) (2024) 16–37, 10.1080/02732173.2023.2279968.

[60] Demski C, Capstick S, Pidgeon N, G Sposato R, Spence A, Experience of extreme weather affects climate change mitigation and adaptation responses, Clim. Change 140 (2) (2017) 149–164, 10.1007/s10584-016-1837-4. 32355377 PMC7175646

[61] Osberghaus D, Demski C, The causal effect of flood experience on climate engagement: evidence from search requests for green electricity, Clim. Change 156 (1–2) (2019) 191–207, 10.1007/s10584-019-02468-9.

[62] Skov T, Cordtz T, Jensen LK, Saugman P, Schmidt K, Theilade P, Modifications of health behaviour in response to air pollution notifications in Copenhagen, Soc. Sci. Med. 33 (5) (1991) 621–626, 10.1016/0277-9536(91)90220-7. 1720575

[63] Bockarjova M, Steg L, Can protection motivation theory predict pro-environmental behavior? Explaining the adoption of electric vehicles in the Netherlands, Glob. Environ. Change 28 (September) (2014) 276–288, 10.1016/j.gloenvcha.2014.06.010.

[64] Arjomandi A, Y Peyman M, Shirzad A, Komendantova N, Kameli E, Hosseinzadeh M, Razavi E, Institutional trust and cognitive motivation toward water conservation in the face of an environmental disaster, Sustainability. 15 (2) (2023) 900, 10.3390/su15020900.

[65] McCaughey JW, Mundir I, Daly P, Mahdi S, Patt A, Trust and distrust of Tsunami vertical evacuation buildings: extending protection motivation theory to examine choices under social influence, Int. J. Disaster Risk Reduct. 24 (September) (2017) 462–473, 10.1016/j.ijdrr.2017.06.016.

[66] Hall MA, Dugan E, Zheng B, Mishra AK, Trust in physicians and medical institutions: what is it, can it be measured, and does it matter? Milbank. Q. 79 (4) (2001) 613–639, 10.1111/1468-0009.00223. 11789119 PMC2751209

[67] Kim J, S Oh S, Confidence, knowledge, and compliance with emergency evacuation, J. Risk. Res 18 (1) (2015) 111–126, 10.1080/13669877.2014.880728.

[68] Zahran S, Brody SD, Grover H, Vedlitz A, Climate change vulnerability and policy support, Soc. Nat. Resour. 19 (9) (2006) 771–789, 10.1080/08941920600835528.

[69] Dvir R, Liu X, Vedlitz A, Exploring public participation modes in government: The case of infrastructure policies, Pub. Manage. Rev. 26 (10) (2024) 2754–2775, 10.1080/14719037.2023.2196550.

[70] Lorenzoni I, Nicholson-Cole S, Whitmarsh L, Barriers perceived to engaging with climate change among the UK public and their policy implications, Glob. Environ. Change 17 (3–4) (2007) 445–459, 10.1016/j.gloenvcha.2007.01.004.

[71] Akerlof K, Maibach EW, Fitzgerald D, Cedeno AY, Neuman A, Do people ‘personally experience’ Global warming, and if so how, and does it matter? Glob. Environ. Change 23 (1) (2013) 81–91, 10.1016/j.gloenvcha.2012.07.006.

[72] Vicente-Molina MA, Fernández-Sainz A, Izagirre-Olaizola J, Does gender make a difference in pro-environmental behavior? The case of the Basque country university students, J. Clean. Prod. 176 (March) (2018) 89–98, 10.1016/j.jclepro.2017.12.079.

[73] Kahlor LA, An augmented risk information seeking model: the case of global warming, Media Psychol. 10 (3) (2007) 414–435, 10.1080/15213260701532971.

[74] Radecki CM, Jaccard J, Perceptions of knowledge, actual knowledge, and information search behavior, J. Exp. Soc. Psychol 31 (2) (1995) 107–138, 10.1006/jesp.1995.1006.

[75] Doiron D, Fiebig DG, Johar M, Suziedelyte A, Does self-assessed health measure health? Appl. Econ. 47 (2) (2014) 180–194, 10.1080/00036846.2014.967382.

[76] La Porte TR, Metlay DS, Hazards and institutional trustworthiness: facing a deficit of trust, Public Adm. Rev. (1996) 341–347. https://www.jstor.org/stable/976375.

[77] Papke LE, Wooldridge JM, Econometric methods for fractional response variables with an application to 401 (k) plan participation rates, J. Appl. Econom 11 (6) (1996) 619–632, 10.1002/(SICI)1099-1255(199611)11:6%3C619::AID-JAE418%3E3.0.CO;2-1.

[78] Betz T, Domestic institutions, trade disputes, and the monitoring and enforcement of international law, Int. Interact. 44 (4) (2018) 631–660, 10.1080/03050629.2018.1407319.

[79] Mizrahi S, Cohen N, Vigoda-Gadot E, Krup DN, Compliance with government policies during emergencies: trust, participation and protective actions, Governance 36 (4) (2023) 1083–1102, 10.1111/gove.12716.

[80] Baron RM, Kenny DA, The moderator–mediator variable distinction in social psychological research: Conceptual, strategic, and statistical considerations, J. Person. Soc. Psychol. 51 (6) (1986) 1173. 10.1037//0022-3514.51.6.1173 3806354

[81] Henderson L, Green C, Making sense of microplastics? Public understandings of plastic pollution, Mar. Pollut. Bull. 152 (March) (2020) 110908, 10.1016/j.marpolbul.2020.110908. 32479284

[82] Liu L.i-Y, Wu W-N, McEntire DA, Six Cs of pandemic Emergency management: a case study of Taiwan’s initial response to the COVID-19 Pandemic, Int. J. Disaster Risk Reduct. 64 (October) (2021) 102516, 10.1016/j.ijdrr.2021.102516. PMC8373854 34426781

[83] Ning L, Niu J, Bi X, Yang C, Liu Z, Wu Q, Liu C, The impacts of knowledge, risk perception, emotion and information on citizens’ protective behaviors during the outbreak of COVID-19: a cross-sectional study in China, BMC. Public Health 20 (1) (2020) 1751, 10.1186/s12889-020-09892-y. 33225934 PMC7681179

[84] Su LYF, Cacciatore MA, Scheufele DA, Brossard D, Xenos MA, Inequalities in scientific understanding: Differentiating between factual and perceived knowledge gaps, Sci. Commun. 36 (3) (2014) 352–378.

[85] Bateman TS, O’Connor K, Felt responsibility and Climate Engagement: distinguishing adaptation from mitigation, Glob. Environ. Change 41 (November) (2016) 206–215, 10.1016/j.gloenvcha.2016.11.001.

